# Poisoning with Arsenic

**Published:** 1846-10

**Authors:** 


*Poisoning with Arsenic.*—MM. Maresk and Lados have ascertained that when a pregnant woman is poisoned with arsenic, the arsenic may be transmitted to the fœtus. In an official investigation of a case of this kind, they discovered traces of the poison in a fœtus at the fourth month. The uterus and the placenta also contained arsenic, but the placenta contained a proportionally larger quantity than the fœtus. The liquor amnii contained no arsenic, at least the quantity, if any, was inappreciable.—*Bull de la Soc. Med. de Grand.—Med. News.*

